# Pelagic photoferrotrophy and iron cycling in a modern ferruginous basin

**DOI:** 10.1038/srep13803

**Published:** 2015-09-08

**Authors:** Marc Llirós, Tamara García–Armisen, François Darchambeau, Cédric Morana, Xavier Triadó–Margarit, Özgül Inceoğlu, Carles M. Borrego, Steven Bouillon, Pierre Servais, Alberto V. Borges, Jean–Pierre Descy, Don E. Canfield, Sean A. Crowe

**Affiliations:** 1 Laboratory of Freshwater Ecology, Research Unit in Environmental and Evolutionary Biology, University of Namur, B-5000 Namur, Belgium; 2 Department of Genetics and Microbiology, Universitat Autònoma de Barcelona, E-08913 Bellaterra, Catalonia, Spain; 3 Ecologie des Systèmes Aquatiques, Université Libre de Bruxelles, B-1050 Brussels, Belgium; 4 Chemical Oceanography Unit, University of Liège, B-4000 Liège, Belgium; 5 Department of Earth and Environmental Sciences, Katholieke Universiteit Leuven, B-3001 Leuven, Belgium; 6 Integrative Freshwater Ecology Group, Center for Advanced Studies of Blanes – Spanish Research Council, E-17300 Blanes, Spain; 7 Group of Molecular Microbial Ecology, Institute of Aquatic Ecology, University of Girona, E-17071 Girona, Catalonia, Spain; 8 Water Quality and Microbial Diversity, Catalan Institute for Water Research, E-17003, Girona, Catalonia, Spain; 9 Institute of Biology, Nordic Center for Earth Evolution, University of Southern Denmark, 5230 Odense, Denmark; 10 Departments of Microbiology and Immunology and Earth, Ocean, and Atmospheric Sciences, University of British Columbia, V6T 1Z3 Vancouver, Canada

## Abstract

Iron-rich (ferruginous) ocean chemistry prevailed throughout most of Earth’s early history. Before the evolution and proliferation of oxygenic photosynthesis, biological production in the ferruginous oceans was likely driven by photoferrotrophic bacteria that oxidize ferrous iron {Fe(II)} to harness energy from sunlight, and fix inorganic carbon into biomass. Photoferrotrophs may thus have fuelled Earth’s early biosphere providing energy to drive microbial growth and evolution over billions of years. Yet, photoferrotrophic activity has remained largely elusive on the modern Earth, leaving models for early biological production untested and imperative ecological context for the evolution of life missing. Here, we show that an active community of pelagic photoferrotrophs comprises up to 30% of the total microbial community in illuminated ferruginous waters of Kabuno Bay (KB), East Africa (DR Congo). These photoferrotrophs produce oxidized iron {Fe(III)} and biomass, and support a diverse pelagic microbial community including heterotrophic Fe(III)-reducers, sulfate reducers, fermenters and methanogens. At modest light levels, rates of photoferrotrophy in KB exceed those predicted for early Earth primary production, and are sufficient to generate Earth’s largest sedimentary iron ore deposits. Fe cycling, however, is efficient, and complex microbial community interactions likely regulate Fe(III) and organic matter export from the photic zone.

Ferruginous water bodies are rare on the modern Earth, yet they are invaluable natural laboratories for exploring the ecology and biogeochemistry of Fe-rich waters extensible to the ferruginous oceans of the Precambrian Eons^1^,^2^,^3^,^4^. One modern ferruginous system, Lake Matano (Indonesia) hosts large populations of anoxygenic phototrophic bacteria implicated in photoferrotrophy due to the scarcity of sulfur substrates^4^. Low light levels and extremely slow growth rates, however, have precluded the direct measurement of photoferrotrophy in its water column^5^. In contrast, recent measurements of Fe-dependent carbon fixation reveal photoferrotrophy in Lake La Cruz (Spain) where photoferrotrophs have been enriched from the water column, but represent a minor fraction of the natural microbial community^6^. Inspired by the emerging evidence for photoferrotrophy in modern environments, we sought a photoferrotroph-dominated ecosystem that could be used to place constraints on the ecology of ancient ferruginous environments.

Kabuno Bay (KB) is a ferruginous sub-basin of Lake Kivu, situated in the heart of East Africa on the border of the Democratic Republic of Congo (DRC) and Rwanda (Supplementary Fig. S1). Lake Kivu is of tectonic origin and is fed by deep-water inflows containing high concentrations of dissolved salts and geogenic gases^7^. KB is separated from the main basin of Lake Kivu by a shallow volcanic sill that restricts water exchange between the basins^7^. KB has a strongly stratified water column with oxic surface waters giving way to anoxic waters below about 10 m (Fig. 1a,b,e,f; Supplementary Fig. S2a,e)^7^. The deep anoxic waters of KB are iron-rich (Fe(II), 0.5M HCl extractable), containing up to 1.2 mM ferrous Fe {Fe(II)}, unlike the deep waters of Lake Kivu’s main basin, which contain abundant hydrogen sulfide (*ca.* 0.3 mM in deep waters)^8^. Fe(II)-rich hydrothermal springs with chemistry matching deep waters of KB are observed within the catchment basin^9^ (Supplementary Table S1), implicating hydrothermal Fe inputs to KB. Oxidation of upward diffusing Fe(II) generates both sharp gradients in dissolved Fe(II) concentration and an accumulation of mixed-valence Fe particles around the oxic-anoxic boundary (i.e., chemocline; Fig. 1b,c,f,g). Reduction of the settling particulate ferric Fe {Fe(III)} to Fe(II) partly closes the Fe-cycle (Fig. 1c,g).

The physical and chemical stratification of the water column is also reflected in microbial community composition. In the oxic sunlit waters (between surface and 10.0 m depth), cyanobacteria (*ca.* 10% of total cell counts by flow cytometry), algae, and heterotrophic bacteria typical of freshwater environments^10^ dominate (Fig. 1d,h; Fig. 2a; Supplementary Table S2). Light, however, penetrates well below these surface waters illuminating the Fe(II)-rich anoxic waters below (Fig. 1d,h). Here, we find a very different microbial community (Fig. 2a; Supplementary Fig. S2b,c,f,g). Anoxygenic photosynthetic green-sulfur bacteria (GSB) dominate in the chemocline where they comprise up to 30% of the total microbial community (Fig. 2b). Concentrations of Bacteriochlorophyll (BChl) *e*, a photosynthetic pigment utilized by brown-coloured, low light adapted GSB^11^,^12^ reach up to *ca.* 235 μg l^−1^ (Fig. 1d,h) and clearly delineate the distribution of GSB in the chemocline waters. Depth-integrated BChl *e* concentrations (130 mg m^−2^) are 10-fold higher than Chlorophyll (Chl) *a* (13 mg m^−2^) concentrations in the upper waters. Analysis of the 16S small subunit rRNA gene reveals that the GSB present in KB are closely related to *Chlorobium* (*Chl.*) *ferrooxidans* strain KoFox (Fig. 2c and Supplementary Fig. S3 and S4). To date, str. KoFox is the sole member of the GSB known to conduct photoferrotrophy^13^ using Fe(II) as electron donor, and lacking the capacity to grow with reduced sulfur species^13^. Such physiology is consistent with the sub-μM concentrations (0–0.6 μM, maximum at 10.5 m; Fig. 1b,f) of reduced sulfur species observed in the illuminated waters of KB.

To directly test for photoferrotrophic activity in KB, we conducted a suite of incubation experiments in which rates of Fe(II) oxidation were measured over time. In the first set of experiments, we incubated water samples between 10.5 and 11.3 m by suspending triplicate glass incubation vessels directly in the water column so that the microbial community would experience near *in situ* light conditions with an average diel illumination of 0.6 μE m^−2^ s^−1^ and a mid-day maximum of 3.2 μE m^−2^ s^−1^. We measured light-dependant Fe(II) oxidation rates up to 100 μmol Fe l^−1^ d^−1^, demonstrating active photoferrotrophic activity in the KB chemocline (Fig. 3a). At 8 × 10^7^ GSB cells l^−1^, cell specific Fe oxidation rates are up to 1.25 pmol cell^−1^ d^−1^. Depth-integrated Fe(II) oxidation rates of 36.8 mmol Fe m^−2^ d^−1^ were computed by taking the mean of the measured rates between 10.5 and 11.3 m, and multiplying by the 0.8 m interval. This Fe(II) oxidation could drive carbon (C) fixation at rates of 9.2 mmol C m^−2^ d^−1^ based on the expected (4:1) relationship between Fe oxidation to C-fixation during photoferrotrophy^14^; nearly the same rate (9 mmol C m^−2^ d^−1^) as measured directly by ^13^C labelling experiments. Rates of photosynthetic C fixation in the chemocline were up to 28% of the production in the oxic suface waters (32 mmol C m^−2^ d^−1^). While cyanobacteria and GSB co-occur in the chemocline, low average Chl *a* concentrations (*ca.* 1.1 μg l^−1^) and BChl *e*:Chl *a* ratios of more than about 100 highlight the dominance of GSB in the ferruginous waters. The importance of photoferrotrophy in the chemocline of KB is underscored by mass balance on the stable C isotope composition of particulate organic matter. Using a simple isotope-mixing model (Supplementary Information) we estimated that 74% ± 13% of the particulate organic carbon pool in the chemocline is derived through anoxygenic photosynthesis by GSB, with a maximum (89%) at 11.25 m. This mass balance reveals that GSB constituted 208 mmol m^−2^ biomass, which together with the light-dependent C fixation rates translates to a GSB biomass turnover time of 23 d.

Fe(III) reduction rates measured in glass vessels kept dark and incubated alongside the light vessels are nearly equivalent (48 mmol m^−2^ d^−1^) to Fe(II) oxidation rates, suggesting a tightly coupled, pelagic Fe-cycle driven by photoferrotrophy, with comparably little net Fe oxidation. Sulfate reduction and potential sulfide oxidation also occurred, but these S-based metabolisms proceed at rates much lower than Fe-reduction and oxidation, respectively (Fig. 3a). This implies that sulfide produced during sulfate reduction plays a small role in reduction of Fe, and most Fe reduction is likely heterotrophic. Fe reduction driven by GSB biomass breakdown is likely reflected by bacterial production rates, which were highest in the illuminated ferruginous waters of the chemocline (Supplementary Fig. S2d). Photoferrotrophy therefore appears to support much of the biogeochemical cycling in the KB chemocline, with primary production of organic matter driving heterotrophic microbial Fe(III) reduction. The rapid GSB turnover rates estimated through our stable isotope mass balance also indicate the effective breakdown of GSB biomass implying that fermentation of this biomass provides substrates (*e.g.*, CH_3_COO^−^, H_2_) to fuel Fe reduction, and possible pelagic heterotrophy with other electron acceptors such as sulfate, or methanogenesis. Both CH_3_COO^−^ and H_2_ can be detected in the KB water column (Fig. S2e).

To more directly test the potential for pelagic Fe cycling, the microbial community was also subjected to alternating light and dark conditions in a second incubation experiment conducted *ex situ* with an inhibitor of oxygenic photosynthesis^15^ (3-(3,4-dichlorophenyl)-1,1-dimethylurea; DCMU; 0.55 mg l^−1^; Fig. 3b) and at light intensities known to support maximum Fe oxidation rates by *Chl. ferrooxidans* str. KoFox (15 μE m^−2^ s^−1^). These *ex situ* Fe(II) oxidation rates are similar to *in situ* rates (115 μmol l^−1^ d^−1^; Fig. 3b), and at 9 × 10^7^ GSB cells l^−1^ in this experiment translate to 1.3 pmol cell^−1^ d^−1^. Fe(III) reduction rates, in contrast, are much lower (44 μmol l^−1^ d^−1^; Fig. 3b), allowing net Fe(II) oxidation of 71 μmol l^−1^ d^−1^ and Fe(III) accumulation (Fig. 3b). These measurements demonstrate that Fe(II) oxidation can outpace the reactions, like fermentation, that degrade GSB biomass and ultimately lead to Fe(III) reduction.

We also isolated the dominant GSB from the water column into axenic culture. Analyses of the small subunit 16S rRNA gene sequence from the axenic culture reveal that the KB GSB isolate is closely related (98.7% of sequence similarity) to *Chl. ferrooxidans* str. KoFox (Fig. 2c and Supplementary Fig. S4) and clusters with the dominant *Chlorobii* 16S rRNA gene sequences recovered from the KB water column. Unlike str. KoFox though, which only grows in co-culture^13^, the KB strain grows in a pure culture. Str. KB is clearly adapted to pelagic growth under low light conditions synthesizing BChl *e* pigments rather than BChl *c* as does str. KoFox^13^, which was isolated from the surface of shallow creek sediments^13^. Detailed pigment analyses show low-light adaptations in KB strain’s light harvesting apparatus, including high abundances of higher alkylated BChl *e* homologs and a lack of the first BChl *e* homolog (Supplementary Fig. S5). These adaptations may be essential for photoferrotrophy under the low light conditions (Fig. 1h) encountered in ferruginous water columns^11^,^16^. Incubation experiments with the KB isolate also demonstrate its capacity to grow photoferrotrophically under low light conditions (*i.e.*, 0.64 μE m^−2^ s^−1^) oxidizing Fe at a rate of 1.4 mmol l^−1^ d^−1^ (Supplementary Fig. S6).

As primary producers in the KB chemocline, photoferrotrophic GSB play a key role supporting and shaping the resident microbial community. This community is taxonomically and functionally diverse with common diversity metrics indicating nearly 3,000 estimated species (Supplementary Table S3), which is comparable to typical modern coastal marine waters or oxygen-minimum zones^17^,^18^. This community is comprised of known heterotrophic Fe(III)-reducers^19^ with members of the *Rhodoferax* genera making up 8% of the OTUs (operational taxonomic units) retrieved. Other community members include micro-aerophillic Fe(II)-oxidizers, sulfate reducers (e.g., *Desulfobacca*, *Desulfomonile*), fermenters (e.g., *Streptococcus*), methanotrophs (e.g., *Methylobacterium*), and methanogens (e.g., *Methanosaeta, GOM_ArcI*), as well as an appreciable fraction (>13%) of taxa belonging to phyla lacking cultured representatives (Supplementary Table S2 and Fig. 2a). Anoxygenic phototrophs in addition to the 15% GSB, include purple sulfur bacteria and *Chloroflexi*, but these combined never exceed 9% of the OTUs retrieved (Fig. 2a). The archaeal community is dominated by methanogens suggesting pelagic methanogenic activity in these ferruginous waters (Supplementary Fig. S2c,g). Overall, the most abundant taxa in the chemocline are involved in C cycling linked to the oxidation and reduction of Fe, but other members almost certainly play key roles in microbial community metabolism and biogeochemical cycling. For example, the presence of sulfate reducers and methanogens directly in the KB water column implies that some organic matter degradation is channelled through sulfate reduction and methanogenesis, thereby escaping remineralization through Fe reduction. By extension, this also requires that some Fe(III) escapes reduction, perhaps through aging and recrystallization to forms less available towards Fe reduction^20^, for subsequent export to underlying sediments. Mixed valence Fe particles at the base of the chemocline indeed are comprised of 20% Fe(II) and 80% Fe(III) (mean redox state of 2.8, Fig 1c), demonstrating the export of ferric iron from the photic zone.

Our observations from KB provide possible insight into the structure and functioning of ancient photoferrotrophic microbial communities thought to have sustained the global C-cycle prior to the evolution and proliferation of oxygenic phototrophs. Rates of photoferrotrophy in the KB water column (3.4 mol C m^−2^ yr^−1^) are within the range of those modelled for global photoferrotrophic production in Earth’s early ferruginous oceans (1.4 mol m^−2^ yr^−1^ based on 5 × 10^14^ mol C yr^−1^ and an ocean area of 3.6 × 10^14^ m^2^)^21^. Previous computations also suggest Fe deposition rates of up to 45 mol m^−2^ yr^−1^ were needed to deposit the largest Precambrian banded iron formations (BIFs)^22^. Net Fe oxidation rates of 0.8 pmol cell^−1^ d^−1^ in KB show that for a photic zone depth of 100 m, photoferrotrophic GSB at low cell densities of 1.7 × 10^3^ cells ml^−1^ could produce up to 50 mol m^−2^ yr^−1^ Fe(III) under modest light conditions, enough to deposit even the largest BIF (i.e., Hammersley Basin; Australia)^22^. Notably, the mean redox state of 2.8 for mixed valence Fe particles exported from the KB photic zone is sufficiently oxidized to explain the Fe(III) component in many BIFs, which have an average Fe redox state of 2.4^23^. In KB, pelagic Fe(III) reduction results from microbial community metabolism illustrating the importance of considering net Fe(II) oxidation rates in photoferrotrophic models of BIF deposition. Photoferrotrophic deposition of BIF then likely requires that rates of Fe(II) oxidation outpace processes like fermentation that ultimately lead to pelagic Fe(III) reduction. Our observations implicate complex interactions between microbial community metabolism and physical and chemical dynamics in the regulation of C and Fe export from ferruginous euphotic zones, but the quantitative nature of these interactions remains uncertain for now. Future work at KB and in other ferruginous water bodies will help tease apart these interactions, and elucidate the microbial controls on biogeochemical cycling in modern and ancient ferruginous waters.

## Methods Summary

Water samples from the water column of Kabuno Bay (1.58º–1.70º S, 29.01º–29.09º E; DR Congo) were collected in February (rainy season, RS) and October (dry season, DS) 2012 and processed for physico-chemical characteristics, microbial abundance, diversity, activity, and cultivation of green sulfur bacteria. Vertical CTD (conductivity, temperature, depth) profiles were measured *in situ* with two multiparametric probes (Hydrolab DS5, OTT Hydromet, Germany; and Sea & Sun CTD90, Sea and Sun Technology, Germany). Photosynthetically Active Radiation (PAR) was measured using a submersible Li-Cor LI-193SA spherical quantum sensor (Lincoln, NE, USA). pH, CH_4_ (methane) concentrations, and stable C isotopic composition (δ^13^C) of particulate organic carbon (POC; δ^13^C-POC) were measured as previously described^24^,^25^. Bacterial production was estimated from tritiated thymidine (^3^H-Thymidine) incorporation rates^25^,^26^. Bulk light and dark inorganic C fixation was measured by NaH^13^CO_3_ incorporation (see supplementary methods for description). Fe speciation was measured using the ferrozine method^27^, while Fe oxidation and reduction rates were determined following changes in Fe speciation over time. Sulfate reduction rates were determined by using the ^35^S radiotracer method^28^. Photosynthetic pigments were analysed by High Performance Liquid Chromatography according to^29^,^30^. Genomic DNA was extracted as previously described^31^ and further subjected to pyrosequencing^32^. *Chlorobi* enrichment cultures were generated by supplementing water with nutrients and Fe. Isolates were obtained through multiple serial dilutions in a defined mineral media^33^. Small aliquots from the isolates were subjected to polymerase chain reaction (PCR) amplification of the 16S rRNA gene, and PCR products sequenced. All *Chlorobi*-retrieved 16S rRNA gene sequences were analysed by means of ARB^34^ loaded with a SILVA 16S rRNA compatible database.

## Additional Information

**How to cite this article**: Llirós, M. *et al.* Pelagic photoferrotrophy iron cycling in a modern ferruginous basin. *Sci. Rep.* **5**, 13803; doi: 10.1038/srep13803 (2015).

## Supplementary Material

Supplementary Information

## Figures and Tables

**Figure 1:**
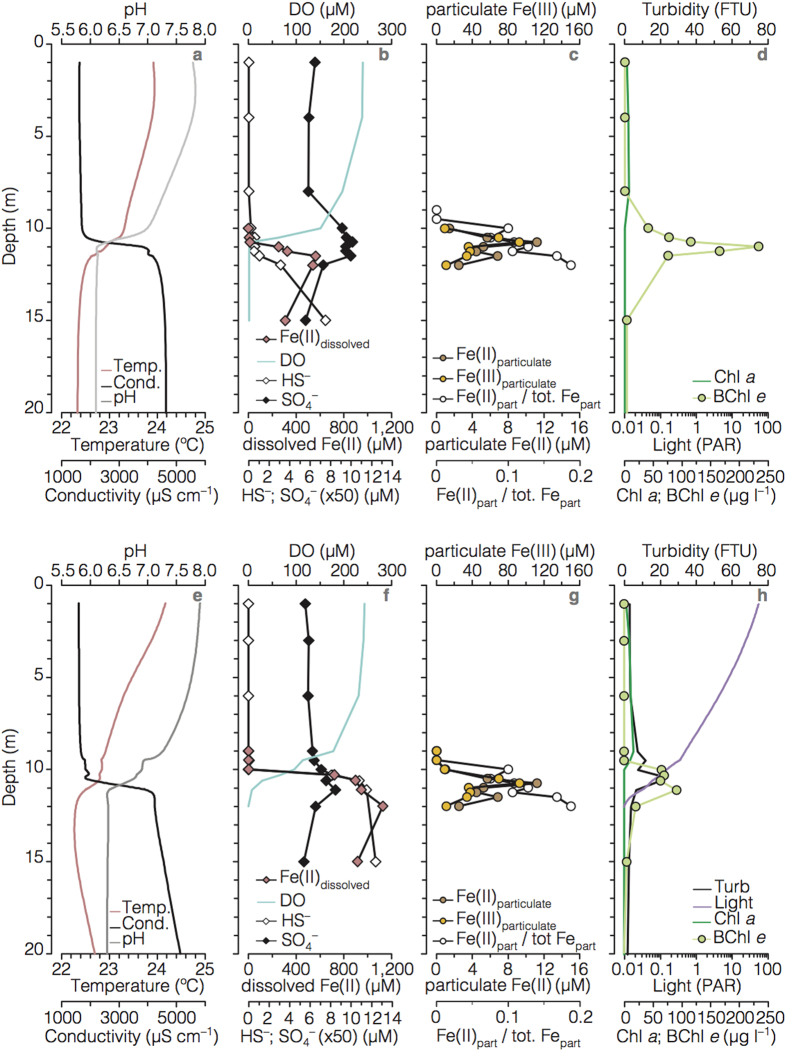
Physical and chemical depth profiles from Kabuno Bay. Data in the upper panels are from the rainy season (RS; February 2012) and lower panels from the dry season (DS; October 2012). (**a**,**e**) temperature (ºC), conductivity (μS cm^−1^), and pH; (**b**,**f**) dissolved oxygen (DO, μM), sulfide (HS^−^, μM), sulfate (SO_4_^−^, μM), and dissolved ferrous Fe (μM); (**c**,**g**) particulate ferrous Fe {Fe(II)} and ferric Fe {Fe(III)} (μM), and ratio of particulate Fe(II) with respect to total particulate Fe (*i.e.*, particulate Fe(II)/[particulate Fe(II) + particulate Fe(III)]); (**d**,**h**) light (% PAR) and turbidity (FTU) profiles, and Chl *a* (μg l^−1^) and intercalibrated BChl *e* concentration (μg l^−1^) measured with multiparametric probes.

**Figure 2:**
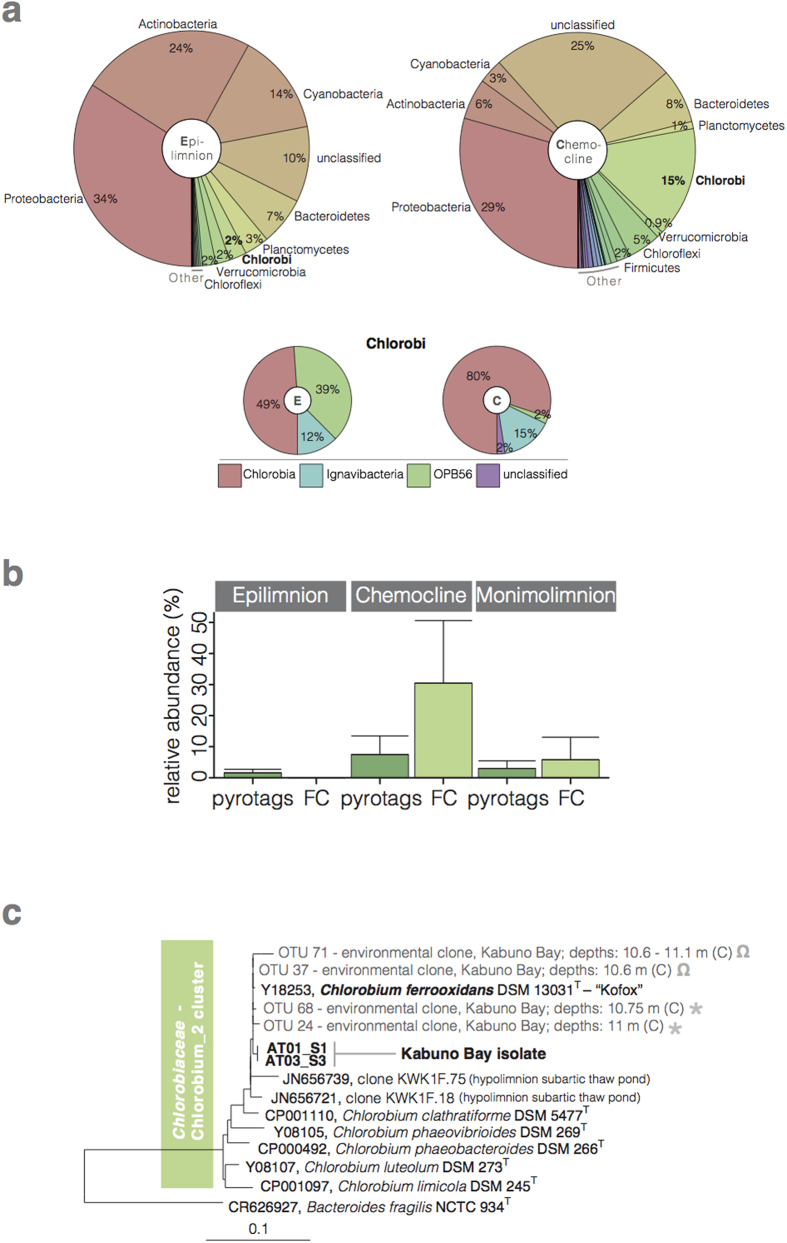
Microbial diversity in Kabuno Bay. (**a**) Pie charts showing relative sequence abundances of retrieved bacterial phyla, with detailed hierarchy for the *Chlorobi* phylum, detected in epilimnetic (left, E), and chemocline (right, C) waters of KB. (**b**) Relative abundances of *Chlorobi* sequences (dark green) retrieved by pyrosequencing (pyrotags) and cell abundances (light green) determined by flow cytometry (FC) from KB water samples. (**c**) 16S rRNA gene phylogenetic tree of the *Chlorobiaceae* including representative OTUs (0.03 cut-off) from those depths with maximum relative abundances of GSB from both the rainy (RS; asterisk) and dry (DS; omega) season water samples, as well as full 16S rRNA gene sequences from the KB isolate. The identifier code for each OTU and the metadata describing the depths and the layers (E for epilimnion, C for chemocline, and M for monimolimnion) where sequences were recovered are also indicated.

**Figure 3:**
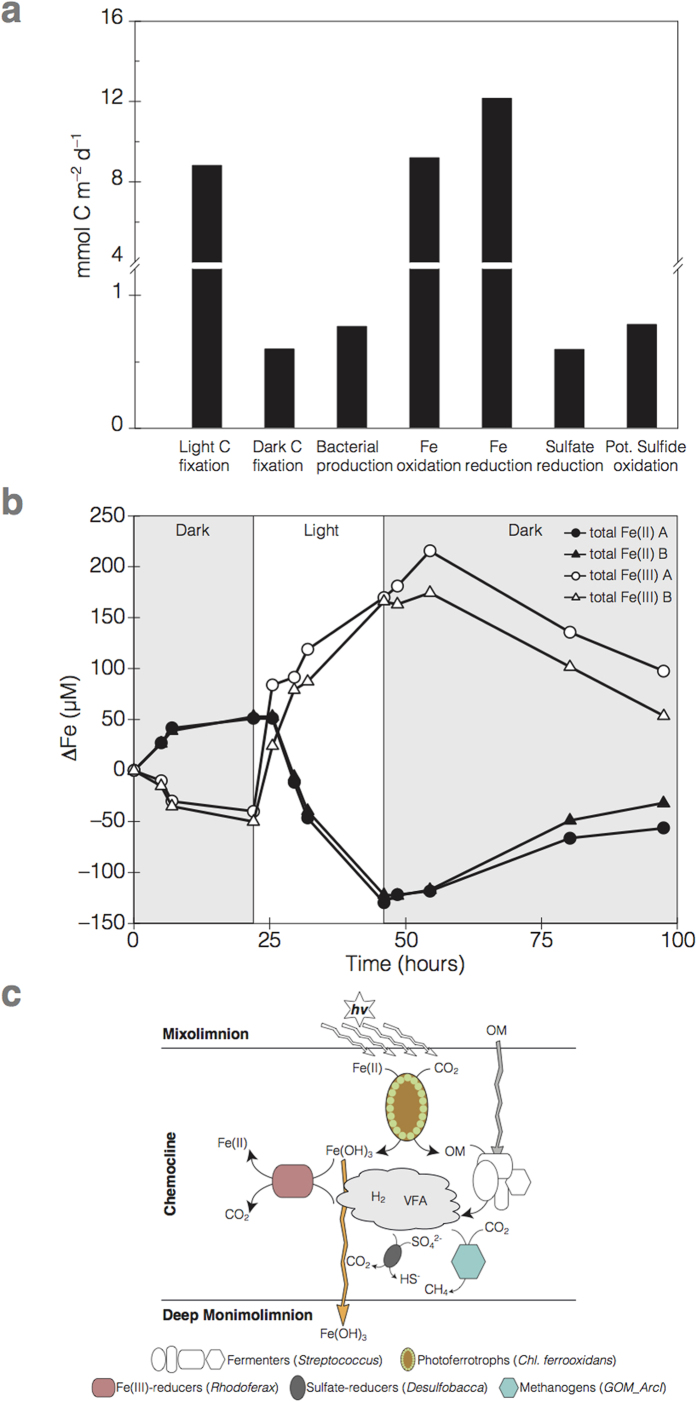
Process rates in Kabuno Bay chemocline. (**a**) depth integrated rates (Carbon normalized; mmol C m^−2^ d^−1^) of: light and dark C fixation; bacterial production (as ^3^H-Thymidine incorporation); Fe oxidation and reduction; sulfate reduction; and potential sulfide oxidation from *in situ* measurements conducted in KB. (**b**) total Fe(II) (black) and total Fe(III) (white) concentrations over time from duplicate vessels incubated *ex situ* through a light (white background) and dark (light grey background) cycle. (**c**) proposed metabolic model for Fe and C cycling in ferruginous waters; legend: *hv*, sunlight; VFA, volatile fatty acids; OM, organic matter.
