# Supplementary material for: Pelagic photoferrotrophy and iron cycling in a modern ferruginous basin
Source: Sci Rep. 2015 Sep 8;5:13803. doi: 10.1038/srep13803 (PMC4562300; doi:10.1038/srep13803)
Supplement: Supplementary Information [file srep13803-s1.pdf]

SUPPLEMENTARY INFORMATION

Pelagic photoferrotrophy and Iron cycling in a modern ferruginous basin

Marc Llirós[1,2,*,†], Tamara García–Armisen[3], François Darchambeau[4], Cédric Morana[5], Xavier Triadó–Margarit[6,7], Özgül Inceoğlu[3], Carles M Borrego[7,8], Steven Bouillon[5], Pierre Servais[3], Alberto V Borges[4], Jean–Pierre Descy[1], Don E Canfield[9] & Sean A Crowe[9,10,*].

[†]Present address: Institute des Sciences de la Vie, Université Catholique de Louvain, Place Croix du Sud 4/5, B-1348 Louvain-La-Neuve, Belgium.

[*]Authors to whom correspondence should be addressed.

SUPPLEMENTARY METHODS

Site description and sampling

Kabuno Bay (KB, 1.58º–1.70º S, 29.01º–29.09º E; DR Congo) is a sub-basin of Lake Kivu located in the East African Rift system. KB has a surface area of 48 km^2 and a maximum depth of 120 m and is permanently stratified. Many physical and chemical characteristics of the basin are related to its isolation from the main lake due to its 150 m wide and 10 m deep connection[1]. Further details on KB and Lake Kivu are published elsewhere[2-11]. Water samples were collected at different depths during two sampling campaigns conducted during 2012 covering both the rainy (RS, February) and the dry (DS, October) seasons using either a battery-driven peristaltic pump connected to a weighted-double conical intake through plastic tubing (for those depths corresponding to the oxic-anoxic transition zone) or a 5-l vertical Niskin bottle (Hydro-Bios; for the rest of the depths). Water samples were processed immediately for chemical analyses or subsequently stored in 4-l plastic containers at 4°C for further processing for molecular microbiology.

Physico-chemical analyses

Vertical depth profiles of temperature, conductivity, pH, and oxygen were measured *in situ* with either Hydrolab DS5 (OTT Hydromet, Germany) or a Sea&Sun CTD90 (Sea and Sun Technology, Germany) multiparametric probes. Photosynthetically Active Radiation (PAR) was measured by means of a Li-Cor LI-193SA spherical quantum sensor apparatus (Lincoln, NE, USA). Fe speciation was measured by ferrozine method[12]. CH_4 concentrations in the collected water samples were determined as previously described[9,13] via the headspace equilibration technique and gas chromatography[14].

Water samples for NOx (*i.e.*, NO_3^- and NO_2^-) analyses were directly passed through 0.22 μm pore size cellulose acetate syringe filters and stored frozen until analyses with no preservative. NO_2^- concentrations were determined following the sulphanilamide coloration method[15], whereas NO_3^- concentrations were measured after vanadium reduction to nitrite and further quantified as previously described[15,16]. Samples for Hydrogen (H_2) determinations were conducted by headspace equilibration technique[17,18]. Furthermore, acetate (CH_3COO^-) concentrations were measured from

frozen water samples collected during the DS sampling campaign by Ion Chromatography using a Dionex Instrument as described here[19].

Samples for the determination of the stable isotope composition ($\delta^{13}C$) of dissolved inorganic carbon (DIC) were collected by gently overfilling 12 ml glass vial (Labco Exetainer) preserved with 20 µl of saturated $HgCl_2$ (final conc., 0.45 mM). For the analysis of $\delta^{13}C$-DIC, a 2 ml helium headspace was created and 100 µl of H_3PO_4 (99%) was added into each vial to convert all DIC species into CO_2. After overnight equilibration, a variable volume of the headspace was injected into an elemental analyser coupled to an isotope ratio mass spectrometer (EA-IRMS; Thermo FlashHT with Thermo DeltaV Advantage). Calibration of $\delta^{13}C$-DIC measurements was performed with certified reference materials (LSVEC and either NBS-19 or IAEA-CO-1). Samples for the determination of the concentration and the stable isotope composition ($\delta^{13}C$) of particulate organic C (POC) were obtained by filtering a known volume of water on pre-combusted (overnight at 450ºC) 0.3 µm pore-size 25 mm diameter glass fiber filters (Advantec GF-75) and kept frozen (–20ºC) until processing. Filters were decarbonated with HCl fumes for 4h, dried, packed in silver cups and analysed on an EA–IRMS (Thermo FlashHT with Thermo DeltaV Advantage). Acetanilide ($\delta^{13}C$ = –27.65‰ ± 0.05) and Leucine ($\delta^{13}C$ = –13.47‰ ± 0.07) were used as standards, and were calibrated in-house against certified standards (IAEA-CH-6). Relative standard deviation for POC measurements was below 5%, and analytical uncertainty for (natural abundance) $\delta^{13}C$-POC measurements was typically better than 0.15‰.

Pigment analyses

Water samples for pigment analyses (2.0 to 3.0 l) were collected and subsequently passed through 47 mm diameter Macherey-Nägel GF5 filters (Düren, Germany). Chlorophyll (Chl)-related pigments were analysed by High Performance Liquid Chromatography (HPLC) from 90% acetone (Fischer Chemical) extracts (10 ml final volume) according to[20,21]. Identification and quantification of pigments were done according to retention times and specific absorption spectra in the eluent solvents. Calibration and Chl *a* quantification were undertaken by using commercial external standards (DHI, Denmark). The complete separation and quantification of

bacteriochlorophyll (BChl) pigments and homologues was determined from the total area of the corresponding peaks and using appropriate molar absorption coefficients[22,23].

Total cell abundances

Total cell abundances were quantified by flow cytometry as previously described[7].

Simple isotope mass balance

The contribution of *Chlorobium*-derived carbon to the POC pool ($\%POC_{Chlorobium}$) at a given depth (z) was estimated via an isotope mass balance approach, as:

$$\%POC_{Chlorobium} = (100 \times \delta^{13}C\text{-}POC_{z} - 100 \times \delta^{13}C\text{-}POC_{ML}) / (\delta^{13}C\text{-}Chlorobium - \delta^{13}C\text{-}POC_{ML})$$

where $\delta^{13}C$-POC_z is the $\delta^{13}C$ signature of the POC at the depth z, $\delta^{13}C$-POC_{ML} (–27.5‰ ± 0.3, n = 3) is the $\delta^{13}C$ signature of the POC in the mixed layer, and $\delta^{13}C$-*Chlorobium* is the theoretical $\delta^{13}C$ signature of the *Chlorobium*, estimated based on the isotope fractionation factor for C fixation by *Chlorobioum* via the rTCA pathway (–12.2‰,[24]) and the measured $\delta^{13}C$-DIC in the chemocline (–5.3‰ ± 0.2, n = 5).

Light and dark CO_2 fixation

Photo- and chemoautotrophic bulk CO_2 fixation rates were quantified in 60 ml glass serum bottles from selected depths covering the oxic-anoxic transition zone of KB during both RS and DS. Serum bottles (eight per depth) were overflowed 3 times, completely filled avoiding bubbles and then capped with butyl stoppers and crimp-sealed with aluminium caps. The incubation set included clear (light) and dark (aluminium foil covered) triplicate bottles. All samples were spiked with 1 ml of a ^{13}C-DIC solution (99.8% $NaH^{13}CO_3$ dissolved in lake water; final concentration 1mM, equivalent to less than 8% of total DIC stock) through the septa. Additionally, one $HgCl_2$-killed bottle for each condition was incubated as a control without biological activity. Each bottle was gently shaken and incubated for 24h at their corresponding depths. After incubation, 40 ml sub-samples were passed through 0.3 μm pore-size glass fiber filters (Advantec GF-75) to trace ^{13}C-DIC incorporation into the POC pool, and 12 ml sub-samples were used to fill Exetainer tubes (LabCo) poisoned with 20 μl of a saturated solution of $HgCl_2$ (final conc., 0.45 mM) to determine the exact ^{13}C-enrichment in the DIC pool in every bottle. Measurement of the $\delta^{13}C$-DIC and $\delta^{13}C$-

POC were carried out as described above. CO_2 fixation rates (µmol l^{-1} d^{-1}) were calculated as previously described[25] from dark and light bottles according to equations (1) and (2):

$$(1)\ \mathrm{DarkCO_2} = [\mathrm{POC_f} \times (\%^{13}\mathrm{C\text{-}POC_f} - \%^{13}\mathrm{C\text{-}POC_i})] \times [t \times (\%^{13}\mathrm{C\text{-}DIC} - \%^{13}\mathrm{C\text{-}POC_i})]^{-1}$$

$$(2)\ \mathrm{LightCO_2} = [(\mathrm{POC_f} \times (\%^{13}\mathrm{C\text{-}POC_f} - \%^{13}\mathrm{C\text{-}POC_i})) \times (t \times (\%^{13}\mathrm{C\text{-}DIC} - \%^{13}\mathrm{C\text{-}POC_i}))^{-1}] - \mathrm{DarkCO_2}$$

where POC_f is the POC concentration at the end of the incubation, $\%^{13}C\text{-}POC_i$ and $\%^{13}C\text{-}POC_f$ are the initial an final percentage of ^{13}C in POC, t is the incubation time and $\%^{13}C\text{-}DIC$ is the percentage of ^{13}C in DIC after the addition of the tracer.

Bacterial Production

Bacterial production was estimated from tritiated thymidine (^{3}H-Thymidine) incorporation rates[26]. Briefly, 20 ml of water were incubated in gas tight serum bottles with ^{3}H-Thy (ca. 80 Ci $mmol^{-1}$; ICN Pharmaceuticals) for two to four hours in the dark at *in situ* temperature and at saturation conditions (ca. 50 nM of ^{3}H-Thy,[27]). After incubation, cold trichloroacetic acid (TCA; 5.0%, fin. conc.) was added in order to stop ^{3}H-Thy incorporation and samples were kept cold until passed through 0.22 µm pore-size cellulose nitrate filters (Sartorius). Radioactivity associated with the filters was estimated by liquid scintillation in a Liquid Scintillation Analyser Tri-Carb 2100TR (Packard). Cell production was calculated from ^{3}H-Thy incorporation rates according to recent calibrations[28].

Fe-oxidation and reduction rate measurements.

Water samples from selected depths corresponding to those with maximum of turbidity and red fluorescence signals were incubated either *in situ* or *ex situ* for Fe-oxidation and reduction rate measurements. *In situ* incubations were conducted by suspending incubation vessels (25 ml glass syringes) at the depths of collection so the microbial community experienced near *in situ* light conditions. Parallel vessels were incubated under light and dark (aluminium foil for shielding) conditions, and changes in Fe-speciation were monitored over time. Fe(II) accumulation rates in the dark syringes were used to calculate Fe reduction rates, whereas the difference in Fe(II) accumulation between light and dark syringes was used to calculate Fe oxidation rates. *Ex situ*

incubations were also conducted in glass syringes (100 ml) that were incubated under 15 $\mu E\ m^{-2}\ s^{-1}$ of light (supplied by 60 W incandescent light bulb) with the addition of DCMU (3-(3,4-dichlorophenyl)-1,1-dimethylurea or DCMU, 0.55 mg l^{-1}), a well-known inhibitor of photosystem II and oxygenic photosynthesis[29,30]. Syringes were subjected to alternating light and dark cycles. Rates of oxidation and reduction were calculated from time course changes in Fe speciation[31,32].

Sulfate reduction rates

In situ sulfate reduction rates were determined using the ^{35}S radiotracer technique[33]

Molecular analyses

Water samples (0.5 to 1.0 l) for nucleic acid extraction were processed, filtered and stored as previously described[34,35]. Genomic deoxyribonucleic acid (DNA) was extracted from 0.22-μm filters and DNA extractions (*ca.* 50 μl at 60 – 250 ng μl^{-1}) from each water depth were subsequently analysed by means of tag-encoded FLX-Titanium amplicon pyrosequencing (TEFAP)[36,37] at Research and Testing Laboratory (Lubbock, TX, USA). The archaeal and bacterial TEFAP (aTEFAP and bTEFAP, respectively) were performed using previously described primers[37].

In order to obtain nearly full-length 16S rRNA gene sequences from pure cultures of *Chlorobi* isolates from KB (see below), DNA was amplified with general bacterial primers (27f – 1492r;[38,39]) and sequenced at external facilities.

Pyrosequencing data analyses

Pyrosequencing data was analysed using Mothur[40]. Briefly data was decompressed and sequencing errors were reduced by trimming flows (i.e., denoising) and sequences by applying the following quality criteria: amplicons shorter than 200 bp in length, reads containing any unresolved nucleotides and more than 8 homopolimers were removed from the archaeal and bacterial pyrosequencing-derived datasets. Subsequently, improved sequences were processed from individual files. Putative chimeras (checked by using Uchime software[41]) and contaminants (understood as those unclassified or misclassified sequences) were removed from our sequence collection files. Afterwards, alpha- and beta-diversity analyses by OTUs and phylogenetic relationships were conducted. For the taxonomic adscription of both aTEFAP and bTEFAP amplicons, the

SEED alignment for archaea and bacteria from the SILVA databases was uploaded in Mothur[40] and a confidence threshold of 80% (bootstrap) was applied. Relative abundances of retrieved phylogenetic groups and hierarchical visualization was performed with Krona tool version 2-4[42].

Phylogenetic analyses of the GSB community present in KB

bTEFAP sequences from KB water samples tentatively belonging to the *Chlorobi* phylum and 16S rRNA gene sequences from KB isolates were subsequently analysed for further taxonomic refinement. All sequences were aligned using the SINA aligner[43] and then imported into the latest SILVA 16S rRNA-ARB-compatible database (SSURef-111_NR_98_04_08_12_opt_v2.arb; http://www.arb-silva.de) in ARB[44]. Two base frequency filters ("Termini" and "pos_var_ssuref:bacteria"; positional variability by parsimony) were applied to exclude highly variable positions before adding sequences to the original database using the "parsimony quick add marked" tool from ARB. Sequence identities of representative OTUs retrieved from water samples with respect to both *Chlorobium ferrooxidans* strain KoFox and KB isolates were determined by pairwise comparisons in BLAST analyses[45].

Culture conditions

Water samples for culturing were collected from chemocline depths. Enrichment cultures were initiated by supplementing lake water with a defined mineral media[46]. Isolates were obtained from these enrichment cultures through multiple serial dilutions.

References of supplementary methods

1. Damas, H. La stratification thermique et chimique des lacs Kivu, Édouard et Ndalaga (Congo Belge). *Annales de la Société Royale Zoologique de Belgique* **68,** 51–68 (1937).
2. Degens, E. T. & Kulbicki, G. Hydrothermal origin of metals in some East African rift lakes. *Mineral Deposita* **8,** 388–404 (1973).
3. Degens, E. T., Herzen, R. P., Wong, H.-K., Deuser, W. G. & Jannasch, H. W. Lake Kivu: structure, chemistry and biology of an East African rift lake. *Geol Rundsch* **62,** 245–277 (1973).
4. Tietze, D. K. *et al.* The genesis of the methane in Lake Kivu (Central Africa). *Geol Rundsch* **69,** 452–472 (1980).
5. Isumbisho, M., Sarmento, H., Kaningini, B., Micha, J.-C. & Descy, J.-P. Zooplankton of Lake Kivu, East Africa, half a century after the Tanganyika sardine introduction. *J Plankton Res* **28,** 971–989 (2006).
6. Sarmento, H., Isumbisho, M. & Descy, J.-P. Phytoplankton ecology of Lake Kivu (eastern Africa). *J Plankton Res* **28,** 815–829 (2006).
7. Sarmento, H. *et al.* Abundance and distribution of picoplankton in tropical, oligotrophic Lake Kivu, eastern Africa. *Freshwater Biol* **53,** 756–771 (2008).
8. Descy, J.-P., Darchambeau, F. & Schmid, M. *Lake Kivu.* 1–169 (Springer, 2012).
9. Borges, A. V. *et al.* Carbon cycling of Lake Kivu (East Africa): net autotrophy in the epilimnion and emission of CO_2 to the atmosphere sustained by geogenic inputs. *PLoS ONE* **9,** e109500 (2014).
10. Pasche, N., Dinkel, C., Müller, B., Schmid, M. & Wehrli, B. Physical and biogeochemical limits to internal nutrient loading of meromictic Lake Kivu. *Limnol. Oceanogr.* **54,** 1863–1873 (2009).
11. Schmid, M., Halbwachs, M., Wehrli, B. & Wüest, A. Weak mixing in Lake Kivu: New insights indicate increasing risk of uncontrolled gas eruption. *Geochem Geophys Geosyst* **6,** 1–11 (2005).
12. Viollier, E., Inglett, P. W., Hunter, K., Roychoudhury, A. N. & Van Cappellen, P. The ferrozine method revisited: Fe(II)/Fe(III) determination in natural waters. *Appl. Geochem.* **15,** 785–790 (2000).
13. Borges, A. V., Abril, G., Delille, B., Descy, J.-P. & Darchambeau, F. Diffusive methane emissions to the atmosphere from Lake Kivu (Eastern Africa). *J. Geophys. Res.* **116,** 1–15 (2011).
14. Weiss, R. F. Determinations of Carbon Dioxide and Methane by Dual Catalyst Flame Ionization Chromatography and Nitrous Oxide by Electron Capture Chromatography. *J Chromatogr Sci* **19,** 611–616 (1981).
15. Rice, E. W., Baird, R. B., Eaton, A. D. & Clesceri, L. S. *Standard Methods for the Examination of Water Wastewater*. (2012).
16. Miranda, K. M., Espey, M. G. & Wink, D. A. A Rapid, Simple Spectrophotometric Method for Simultaneous Detection of Nitrate and Nitrite. *Nitric Oxide* **5,** 62–71 (2001).
17. Lovley, D. R. & Goodwin, S. Hydrogen concentrations as an indicator of the predominant terminal electron-accepting reactions in aquatic sediments. *Geochim Cosmochim Acta* **52,** 2993–3003 (1988).
18. Hoehler, T. M., Alperin, M. J., Albert, D. B. & Martens, C. S. Thermodynamic control on hydrogen concentrations in anoxic sediments. *Geochim Cosmochim Acta* **62,** 1745–1756 (1998).
19. Vandieken, V., Finke, N. & Thamdrup, B. Hydrogen, acetate, and lactate as

electron donors for microbial manganese reduction in a manganese-rich coastal marine sediment. *FEMS Microbiology Ecology* **87,** 733–745 (2014).

20. Descy, J.-P., Higgins, H. W., Mackey, D. J., Hurley, J. P. & Frost, T. M. Pigment ratios and phytoplankton assessment in northern Wisconsin lakes. *J. Phycol.* **36,** 274–286 (2000).
21. Wright, S. W. *et al.* Improved HPLC method for the analysis of chlorophylls and carotenoids from marine phytoplankton. *Mar Ecol Prog Ser* **77,** 183–196 (1991).
22. Borrego, C. M. & Garcia-Gil, L. J. Separation of bacteriochlorophyll homologues from green photosynthetic sulfur bacteria by reversed-phase HPLC. *Photosynth. Res.* **41,** 157–164 (1994).
23. Borrego, C. M., Arellano, J. B., Abella Ametller, C. A., Gillbro, T. & Garcia-Gil, J. The molar extinction coefficient of bacteriochlorophyll e and the pigment stoichiometry in Chlorobium phaeobacteroides. *Photosynth. Res.* **60,** 257–264 (1999).
24. Sirevåg, R., Buchanan, B. B., Berry, J. A. & Troughton, J. H. Mechanisms of CO2 fixation in bacterial photosynthesis studied by the carbon isotope fractionation technique. *Arch. Microbiol.* **112,** 35–38 (1977).
25. Hama, T. *et al.* Measurement of photosynthetic production of a marine phytoplankton population using a stable ^{13}C isotope. *Mar. Biol.* **73,** 31–36 (1983).
26. Fuhrman, J. A. & Azam, F. Thymidine incorporation as a measure of heterotrophic bacterioplankton production in marine surface waters: Evaluation and field results. *Mar. Biol.* **66,** 109–120 (1982).
27. Servais, P. Bacterioplanktonic biomass and production in the river Meuse (Belgium). *Hydrobiologia* **174,** 99–110 (1989).
28. Stenuite, S. *et al.* Abundance and production of bacteria, and relationship to phytoplankton production, in a large tropical lake (Lake Tanganyika). *Freshwater Biol* **54,** 1300–1311 (2009).
29. Steenbergen, C. L. M. & van den Hoven, P. A note on the measurement of production of phototrophic bacteria in deep layers. *Arch. Hydrobiol. Beih. Ergebn. Limnol.* **34,** 349–355 (1990).
30. Jørgensen, B. B., Kuenen, J. G. & Cohen, Y. Microbial transformations of sulfur compounds in a stratified lake (Solar Lake, Sinai). *Limnol. Oceanogr.* **24,** 799–822 (1979).
31. Crowe, S. A. *et al.* Photoferrotrophs thrive in an Archean Ocean analogue. *Proc. Natl. Acad. Sci. USA* **105,** 15938–15943 (2008).
32. Crowe, S. A. *et al.* The methane cycle in ferruginous Lake Matano. *Geobiology* **9,** 61–78 (2011).
33. Fossing, H. & Jørgensen, B. B. Measurement of bacterial sulfate reduction in sediments – Evaluation of a single-step Chromium reduction method. *Biogeochemistry* **8,** 205–222 (1989).
34. Llirós, M. *et al.* Vertical distribution of ammonia-oxidizing crenarchaeota and methanogens in the epipelagic waters of Lake Kivu (Rwanda-Democratic Republic of the Congo). *Appl Environ Microbiol* **76,** 6853–6863 (2010).
35. Llirós, M., Casamayor, E. O. & Borrego, C. M. High archaeal richness in the water column of a freshwater sulfurous karstic lake along an interannual study. **66,** 331–342 (2008).
36. Dowd, S. E. *et al.* Evaluation of the bacterial diversity in the feces of cattle using 16S rDNA bacterial tag-encoded FLX amplicon pyrosequencing (bTEFAP). *BMC Microbiol* **8,** 125 (2008).

37. Shah, V. *et al.* Bacterial and archaea community present in the Pine Barrens Forest of Long Island, NY: unusually high percentage of ammonia oxidizing bacteria. *PLoS ONE* **6,** e26263 (2011).
38. Suzuki, M. & Giovannoni, S. J. Bias Caused by Template Annealing in the Amplification of Mixtures of 16S rRNA Genes by PCR. *Appl Environ Microbiol* **62,** 625–630 (1996).
39. Stahl, D. A. & Amann, R. in *Nucleic Acid Techniques in Bacterial Systematics* (eds. Stackebrandt, E. & Goodfellow, M.) 205–248 (John Wiley & Sons, Inc., 1991).
40. Schloss, P. D. *et al.* Introducing mothur: open-source, platform-independent, community-supported software for describing and comparing microbial communities. *Appl Environ Microbiol* **75,** 7537–7541 (2009).
41. Edgar, R. C., Haas, B. J., Clemente, J. C., Quince, C. & Knight, R. UCHIME improves sensitivity and speed of chimera detection. *Bioinformatics* **27,** 2194–2200 (2011).
42. Ondov, B. D., Bergman, N. H. & Phillippy, A. M. Interactive metagenomic visualization in a Web browser. *BMC Bioinformatics* **12,** 385 (2011).
43. Pruesse, E., Peplies, J. & Glöckner, F. O. SINA: accurate high-throughput multiple sequence alignment of ribosomal RNA genes. *Bioinformatics* **28,** 1823–1829 (2012).
44. Ludwig, W. *et al.* ARB: a software environment for sequence data. *Nucleic Acids Res.* **32,** 1363–1371 (2004).
45. Altschul, S. F., Gish, W., Miller, W., Myers, E. W. & Lipman, D. J. Basic local alignment search tool. *J Mol Biol* **215,** 403–410 (1990).
46. Hegler, F., Posth, N. R., Jiang, J. & Kappler, A. Physiology of phototrophic iron(II)-oxidizing bacteria: implications for modern and ancient environments. **66,** 250–260 (2008).

Captions to Supplementary Figures

Supplementary Figure S1. Sample location map. Map showing the location of Kabuno Bay, Sake river and hydrothermal spring sampling sites (red dots). Maps were generated by hand-drawing using Adobe Illustrator® software.

Supplementary Figure S2. Kabuno Bay vertical profiles. Data in the upper panels are from the rainy season (RS; February 2012) and lower panels from the dry season (DS; October 2012). **a** and **c**: CH_4 (light blue square), H_2 (grey dots), NO_x (*i.e.*, $NO_2^- + NO_3^-$) (yellow dots) and CH_3COO^- (white squares) concentrations; **b** and **f**: relative abundance of bacterial OTUs grouped by putative activity (i.e., oxygenic phototrophs (Ox PhS, brown dots), anoxygenic phototrophs (Anox PhS (GSB), light green dots), Fe or Mn related bacterial OTUs (red triangles)); **c** and **g**: relative abundance of archaeal OTUs grouped by putative activity (i.e., ammonia oxidizing Archaea (AOA, yellow triangles), acetoclastic methanogens (light blue diamonds) and hydrogenotrophic methanogens (light blue dots)); **d**: ^{3}H-Thymidine uptake (nM ^{3}H-Thymidine h^{-1}, white triangles).

Supplementary Figure S3. *Chlorobi* and related groups diversity. Collapsed 16S rRNA gene phylogenetic tree of the *Chlorobi* and related groups members detected in KB (grey shadow). The number of sequences assigned to each phylogenetic clade and the relative abundance (pie charts) of the retrieved sequences in each clade is indicated. The tree includes sequences retrieved by pyrosequencing from all depths and seasons (RS and DS), and also full 16S rRNA gene sequences from our KB pure culture. Pie charts depict the OTUs retrieved from each water compartment (E stands for epilimnion, C for chemocline and M for monimolimnion) and size is proportional to the number of sequences (log transformed) from the OTUs ascribed to each cluster. The scale bar indicates 0.10 fixed point mutation per nucleotide position.

Supplementary Figure S4. Kabuno Bay GSB sequence similarities. Box plots summarizing sequence identities of representative KB OTUs with respect to both *Chl. ferrooxidans* str. KoFox and to KB isolate.

Supplementary Figure S5. Pigment analyses. Example of a HPLC chromatogram from a natural KB water sample (10.6 m depth, DS) showing the main BChl *e* homologues (farnesyl esterified), the secondary homologues (non-farnesyl esterified) and carotenoids (mainly isorenieratene and ß-isorenieratene).

Supplementary Figure S6. Fe oxidation rates from Kabuno Bay isolate. Fe(II) oxidation rates for KB isolate incubated under high (black diamonds, 14.5 μE m^{-2} s^{-1}) and low (grey diamonds, 0.64 μE m^{-2} s^{-1}) light intensities.

Supplementary table 1. **Physico-chemical characteristics of Kabuno Bay and surrounding water inputs.** Comparison of main physico-chemical parameters measured in the Sake river and hydrothermal spring near Sake Bay in comparison to KB.

	**Kabuno Bay**			**Sake River**	**Hydrothermal spring**
GPS coordinates	1º37.252' S, 29º2.976' E			1°34.078' S, 29°3.236' E	1°33.744' S, 29°2.836' E
Depth (m)	Chemocline<br>10.5 – 11.5	65.0	Deep waters<br>100.0	Surface<br>0.1	Surface<br>0.1
Altitude (m.a.s.l.)	1,463	1,463	1,463	1,515	1,485
Temperature (ºC)	22.96	24.07	24.78	18.55	29.45
pH	6.25	6.1	6.1	6.05	6.32
Dissolved oxygen (mg l^{-1})	2.5	0.0	0.0	1.9	0
Conductivity (mS cm^{-1})	3.27	6.58	7.48	1.29	5.20
Cl^- (mM)	Na[a]	2.1	Na	Na	15.3
SO_4^{2-} (µM)	485,61	0.7	Na	Na	1,574
Fe (II) (µM)	289,79	689	422	0	410

[a]Na, not analysed.

Sup Table 2 - Taxonomic afiliations Kabuno Bay

				February 2012												October 2012										
**Domain**	**Phylum**	**Class**	**Order**	**total seqs**	**1**	**4**	**10**	**10.5**	**10.75**	**11**	**11.25**	**11.5**	**12**	**15**	**30**	**total seqs**	**1**	**6**	**9**	**9.5**	**10**	**10.3**	**10.6**	**11.1**	**15**	**30**
k__Bacteria	p__Actinobacteria			775	215	356	92	12	3	8	9	16	17	45	2	2401	450	331	354	532	424	8	103	112	85	2
k__Bacteria	p__Armatimonadetes			112	0	5	2	3	6	5	14	12	33	22	10	68	1	1	4	5	2	0	1	54	0	0
k__Bacteria	p__Bacteroidetes			861	54	81	52	48	61	51	85	115	103	137	74	1149	170	86	71	131	173	1	73	418	23	3
k__Bacteria	p__Bacteroidetes	c__Bacteroidia		15	0	0	5	3	0	0	2	1	0	3	1	27	0	0	0	0	0	0	16	11	0	0
k__Bacteria	p__Bacteroidetes	c__Flavobacteria		35	9	10	7	0	0	0	3	1	1	3	1	145	26	5	19	29	59	0	5	2	0	0
k__Bacteria	p__Bacteroidetes	c__Sphingobacteria		152	25	41	11	4	4	5	10	18	10	19	5	400	97	50	32	69	49	1	27	59	14	2
k__Bacteria	p__Bacteroidetes	unclassified		659	20	30	29	41	57	46	70	95	92	112	67	577	47	31	20	33	65	0	25	346	9	1
k__Bacteria	p__Chlorobi			1916	8	8	108	265	326	320	229	208	109	183	152	811	23	15	29	42	52	0	122	528	0	0
k__Bacteria	p__Chlorobi	c__Chlorobia		1462	0	1	81	225	274	263	170	143	60	125	120	575	1	0	14	17	26	0	107	410	0	0
k__Bacteria	p__Chlorobi	c__Ignavibacteria		356	0	0	23	35	41	47	44	56	40	43	27	123	0	0	2	4	2	0	15	100	0	0
k__Bacteria	p__Chlorobi	c__OPB56		39	8	7	4	0	0	2	5	4	5	3	1	102	22	15	13	21	24	0	0	7	0	0
k__Bacteria	p__Chlorobi	unclassified		59	0	0	0	5	11	8	10	5	4	12	4	11	0	0	0	0	0	0	0	11	0	0
k__Bacteria	p__Chloroflexi			824	9	13	26	64	33	60	83	88	132	251	65	444	49	29	22	30	83	0	7	224	0	0
k__Bacteria	p__Cyanobacteria			910	243	337	59	19	25	20	18	24	60	48	57	1052	223	168	126	205	164	0	67	97	2	0
k__Bacteria	p__Firmicutes			46	2	2	1	0	3	0	0	3	2	2	31	341	4	5	7	11	0	126	76	20	49	43
k__Bacteria	p__Nitrospirae			102	0	0	3	5	5	6	17	29	10	11	16	29	0	0	0	1	3	0	0	25	0	0
k__Bacteria	p__Planctomycetes			136	33	32	10	3	3	1	7	9	23	2	13	360	134	32	35	54	50	0	14	41	0	0
k__Bacteria	p__Proteobacteria			2945	176	272	601	214	200	128	205	241	231	384	293	5259	646	424	438	759	941	87	671	1115	97	81
k__Bacteria	p__Proteobacteria	c__Alphaproteobacteria		501	92	136	48	28	38	21	27	23	22	42	24	1623	346	221	190	349	269	7	117	114	10	0
k__Bacteria	p__Proteobacteria	c__Alphaproteobacteria	o__Caulobacterales	15	2	1	3	1	0	0	0	1	5	1	1	123	11	10	15	28	5	0	20	34	0	0
k__Bacteria	p__Proteobacteria	c__Alphaproteobacteria	o__Rhizobiales	16	3	3	2	1	0	2	3	1	0	1	0	54	6	0	4	14	2	1	8	19	0	0
k__Bacteria	p__Proteobacteria	c__Alphaproteobacteria	o__Rhodobacterales	143	5	3	6	22	32	16	15	14	4	11	15	76	6	1	0	14	16	6	18	5	10	0
k__Bacteria	p__Proteobacteria	c__Alphaproteobacteria	o__Rhodospirillales	62	6	12	8	3	3	2	4	1	3	16	4	113	14	11	12	18	29	0	13	16	0	0
k__Bacteria	p__Proteobacteria	c__Alphaproteobacteria	o__Rickettsiales	220	73	109	16	0	1	0	3	2	5	10	1	1034	276	186	140	211	181	0	35	5	0	0
k__Bacteria	p__Proteobacteria	c__Alphaproteobacteria	o__Sphingomonadales	18	0	0	7	1	0	0	1	2	4	0	3	100	5	4	2	33	3	0	22	31	0	0
k__Bacteria	p__Proteobacteria	c__Alphaproteobacteria	unclassified	27	3	8	6	0	2	1	1	2	1	3	0	123	28	9	17	31	33	0	1	4	0	0
k__Bacteria	p__Proteobacteria	c__Betaproteobacteria		1294	57	86	268	139	136	85	84	75	47	103	214	1550	137	85	117	194	337	65	286	268	40	21
k__Bacteria	p__Proteobacteria	c__Betaproteobacteria	o__Burkholderiales	706	22	42	142	93	61	43	39	39	28	45	152	865	65	47	48	100	153	62	179	150	40	21
k__Bacteria	p__Proteobacteria	c__Betaproteobacteria	o__Gallionellales	50	0	0	26	8	0	0	0	0	2	14	0	135	0	1	6	11	50	0	31	36	0	0
k__Bacteria	p__Proteobacteria	c__Betaproteobacteria	o__Hydrogenophilales	0	0	0	0	0	0	0	0	0	0	0	0	1	0	0	0	0	0	1	0	0	0	0
k__Bacteria	p__Proteobacteria	c__Betaproteobacteria	o__Methylophilales	71	6	8	44	2	2	0	1	0	1	7	0	177	17	6	21	26	69	0	28	10	0	0
k__Bacteria	p__Proteobacteria	c__Betaproteobacteria	o__Neisseriales	1	0	0	0	0	0	0	0	1	0	0	0	0	0	0	0	0	0	0	0	0	0	0
k__Bacteria	p__Proteobacteria	c__Betaproteobacteria	o__Rhodocyclales	109	0	0	23	12	8	10	13	8	7	17	11	87	0	0	5	6	25	1	17	33	0	0
k__Bacteria	p__Proteobacteria	c__Betaproteobacteria	unclassified	357	29	36	33	24	65	32	31	27	9	20	51	285	55	31	37	51	40	1	31	39	0	0
k__Bacteria	p__Proteobacteria	c__Deltaproteobacteria		471	7	5	12	13	15	14	29	70	92	180	34	541	14	12	19	29	47	0	16	404	0	0
k__Bacteria	p__Proteobacteria	c__Deltaproteobacteria	o__Bdellovibrionales	1	0	0	0	0	0	0	0	1	0	0	0	8	3	2	0	3	0	0	0	0	0	0
k__Bacteria	p__Proteobacteria	c__Deltaproteobacteria	o__Desulfobacterales	5	0	0	0	0	0	0	1	0	4	0	0	12	0	0	0	0	0	0	1	11	0	0
k__Bacteria	p__Proteobacteria	c__Deltaproteobacteria	o__Desulfuromonadales	12	0	1	5	0	0	0	0	1	1	4	0	22	0	0	0	4	7	0	6	5	0	0
k__Bacteria	p__Proteobacteria	c__Deltaproteobacteria	o__MIZ46	13	5	4	3	0	1	0	0	0	0	0	0	44	9	7	16	11	1	0	0	0	0	0
k__Bacteria	p__Proteobacteria	c__Deltaproteobacteria	o__Myxococcales	6	2	0	3	0	1	0	0	0	0	0	0	53	2	1	2	7	29	0	0	12	0	0
k__Bacteria	p__Proteobacteria	c__Deltaproteobacteria	o__Syntrophobacterales	371	0	0	0	9	12	14	26	58	78	150	24	314	0	0	1	2	7	0	5	299	0	0
k__Bacteria	p__Proteobacteria	c__Deltaproteobacteria	unclassified	63	0	0	1	4	1	0	2	10	9	26	10	88	0	2	0	2	3	0	4	77	0	0
k__Bacteria	p__Proteobacteria	c__Epsilonproteobacteria		17	0	0	1	0	0	0	0	2	2	12	0	98	0	0	0	3	2	0	52	41	0	0
k__Bacteria	p__Proteobacteria	c__Epsilonproteobacteria	o__Campylobacterales	17	0	0	1	0	0	0	0	2	2	12	0	98	0	0	0	3	2	0	52	41	0	0
k__Bacteria	p__Proteobacteria	c__Gammaproteobacteria		545	15	26	229	29	7	6	61	65	58	31	18	1238	118	86	90	148	229	15	188	257	47	60
k__Bacteria	p__Proteobacteria	c__Gammaproteobacteria	o__Aeromonadales	2	0	2	0	0	0	0	0	0	0	0	0	1	1	0	0	0	0	0	0	0	0	0
k__Bacteria	p__Proteobacteria	c__Gammaproteobacteria	o__Alteromonadales	12	3	1	0	0	0	0	3	3	2	0	0	42	11	5	2	1	2	0	18	3	0	0
k__Bacteria	p__Proteobacteria	c__Gammaproteobacteria	o__Chromatiales	33	5	12	4	1	2	0	1	0	5	1	2	72	14	19	14	18	4	0	0	3	0	0
k__Bacteria	p__Proteobacteria	c__Gammaproteobacteria	o__Legionellales	14	0	1	2	0	2	1	1	0	1	1	5	56	11	3	6	17	10	0	0	9	0	0
k__Bacteria	p__Proteobacteria	c__Gammaproteobacteria	o__Methylococcales	42	0	0	19	5	1	0	0	4	1	12	0	67	0	0	5	3	35	0	9	15	0	0
k__Bacteria	p__Proteobacteria	c__Gammaproteobacteria	o__Oceanospirillales	1	1	0	0	0	0	0	0	0	0	0	0	4	0	0	3	0	0	1	0	0	0	0
k__Bacteria	p__Proteobacteria	c__Gammaproteobacteria	o__Pasteurellales	0	0	0	0	0	0	0	0	0	0	0	0	9	0	0	0	0	0	0	2	0	0	7
k__Bacteria	p__Proteobacteria	c__Gammaproteobacteria	o__Pseudomonadales	191	1	3	24	7	0	3	52	53	41	1	6	636	63	53	53	82	56	0	83	158	40	48
k__Bacteria	p__Proteobacteria	c__Gammaproteobacteria	o__Xanthomonadales	11	0	0	3	1	0	2	0	1	0	0	4	81	0	0	0	7	1	5	41	15	7	5
k__Bacteria	p__Proteobacteria	c__Gammaproteobacteria	unclassified	239	5	7	177	15	2	0	4	4	8	16	1	270	18	6	7	20	121	9	35	54	0	0
k__Bacteria	p__Proteobacteria	unclassified	unclassified	117	5	19	43	5	4	2	4	6	10	16	3	209	31	20	22	36	57	0	12	31	0	0
k__Bacteria	p__Spirochaetes			45	0	1	0	0	1	0	4	7	14	17	1	75	0	0	0	0	1	0	2	71	0	1
k__Bacteria	p__Verrucomicrobia			80	14	20	11	2	3	0	3	5	5	13	4	239	41	10	30	60	31	0	24	43	0	0
k__Bacteria	other Cult phyla			55	2	6	3	4	1	1	4	7	5	10	12	73	5	2	5	7	3	0	3	30	0	18
k__Bacteria	Cand. Uncult phyla			301	0	0	9	15	22	26	50	59	62	38	20	327	10	4	13	25	42	0	41	192	0	0
k__Bacteria	unclassified			2967	63	126	95	164	177	214	295	468	467	661	237	2924	273	93	111	247	309	1	105	1785	0	0

Supplementary table 3. **Microbial richness and diversity in Kabuno Bay.** Archaeal and bacterial richness and diversity estimates based on pyrosequencing analyses from rainy (RS) and dry (DS) seasons in KB.

Water compartment	# OTUs	Coverage	Richness Chao1	Diversity Shannon
February – RS				
Archaea				
Epilimnion	12	0.99	17	0.3
Chemocline	45	0.99	88	0.7
Monimolimnion	48	0.99	57	1.4
Bacteria				
Epilimnion	776	0.85	1,825	5.4
Chemocline	1,095	0.86	2,837	5.3
Monimolimnion	1,225	0.82	2,859	6.2
October – DS				
Archaea				
Epilimnion	2	–	3	0.7
Chemocline	33	0.98	57	1.5
Monimolimnion	8	0.99	10	0.6
Bacteria				
Epilimnion	109	0.90	2,674	5.2
Chemocline	1,917	0.87	4,784	6.4
Monimolimnion	56	0.94	106	3.3

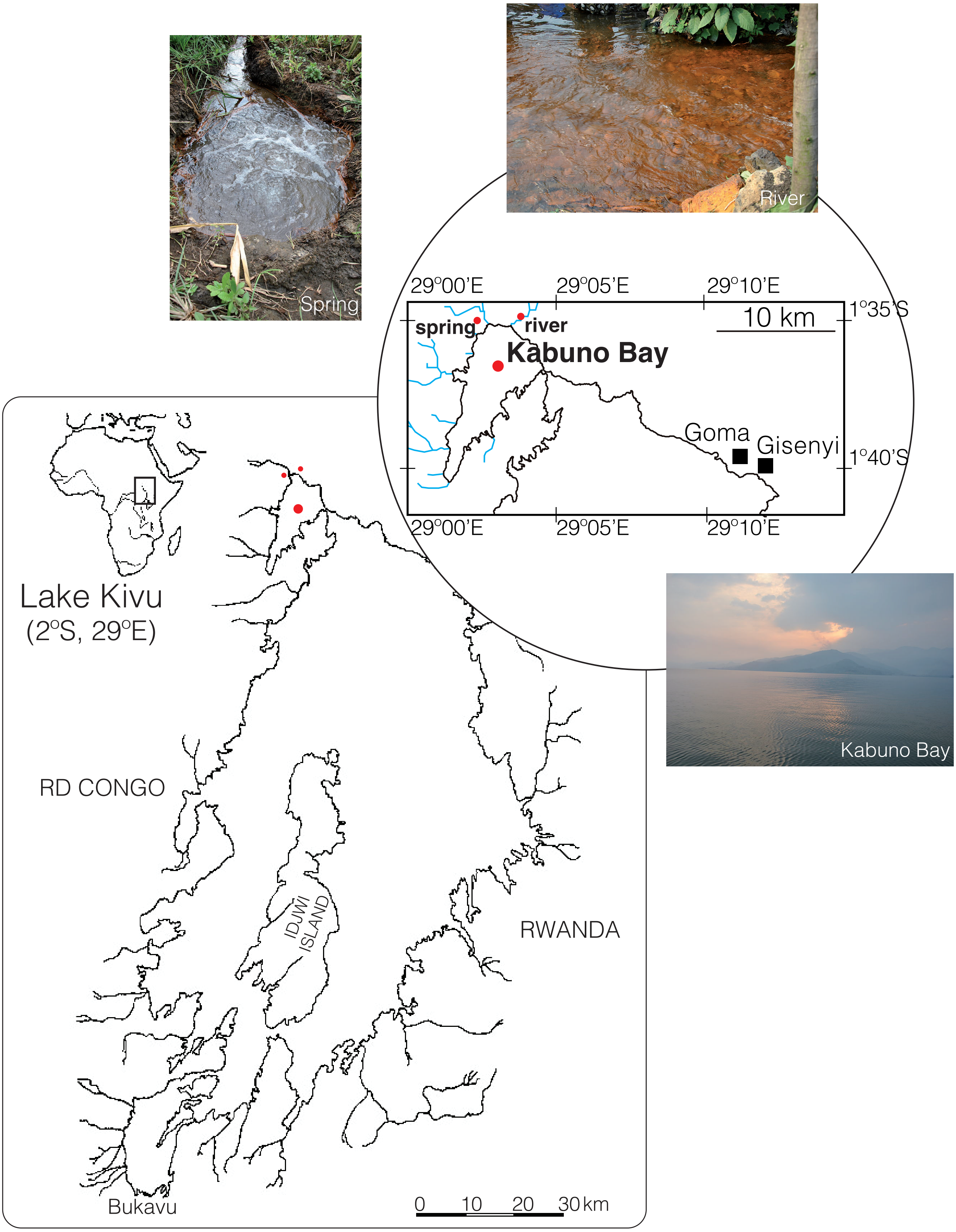
Spring
River
29°00'E
29°05'E
29°10'E
1°35'S
1°40'S
10 km
spring
river
Kabuno Bay
Goma
Gisenyi
Kabuno Bay
Lake Kivu
(2°S, 29°E)
RD CONGO
IDJWI ISLAND
RWANDA
Bukavu
0 10 20 30 km

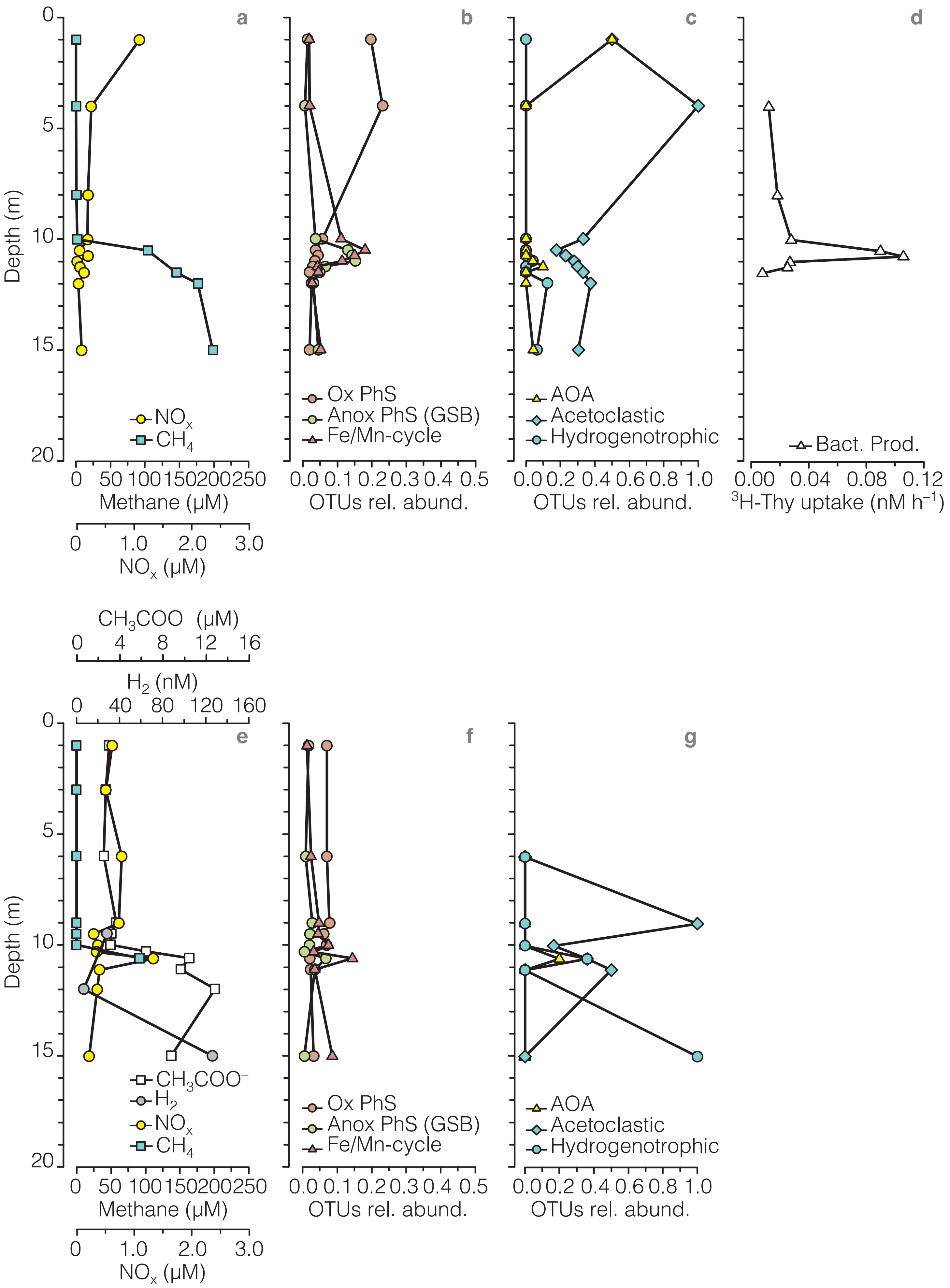

a
b
c
d
Depth (m)
NOx
CH4
Ox PhS
Anox PhS (GSB)
Fe/Mn-cycle
AOA
Acetoclastic
Hydrogenotrophic
Bact. Prod.
Methane (µM)
NOx (µM)
OTUs rel. abund.
3H-Thy uptake (nM h−1)
CH3COO− (µM)
H2 (nM)
e
f
g
CH3COO−
H2

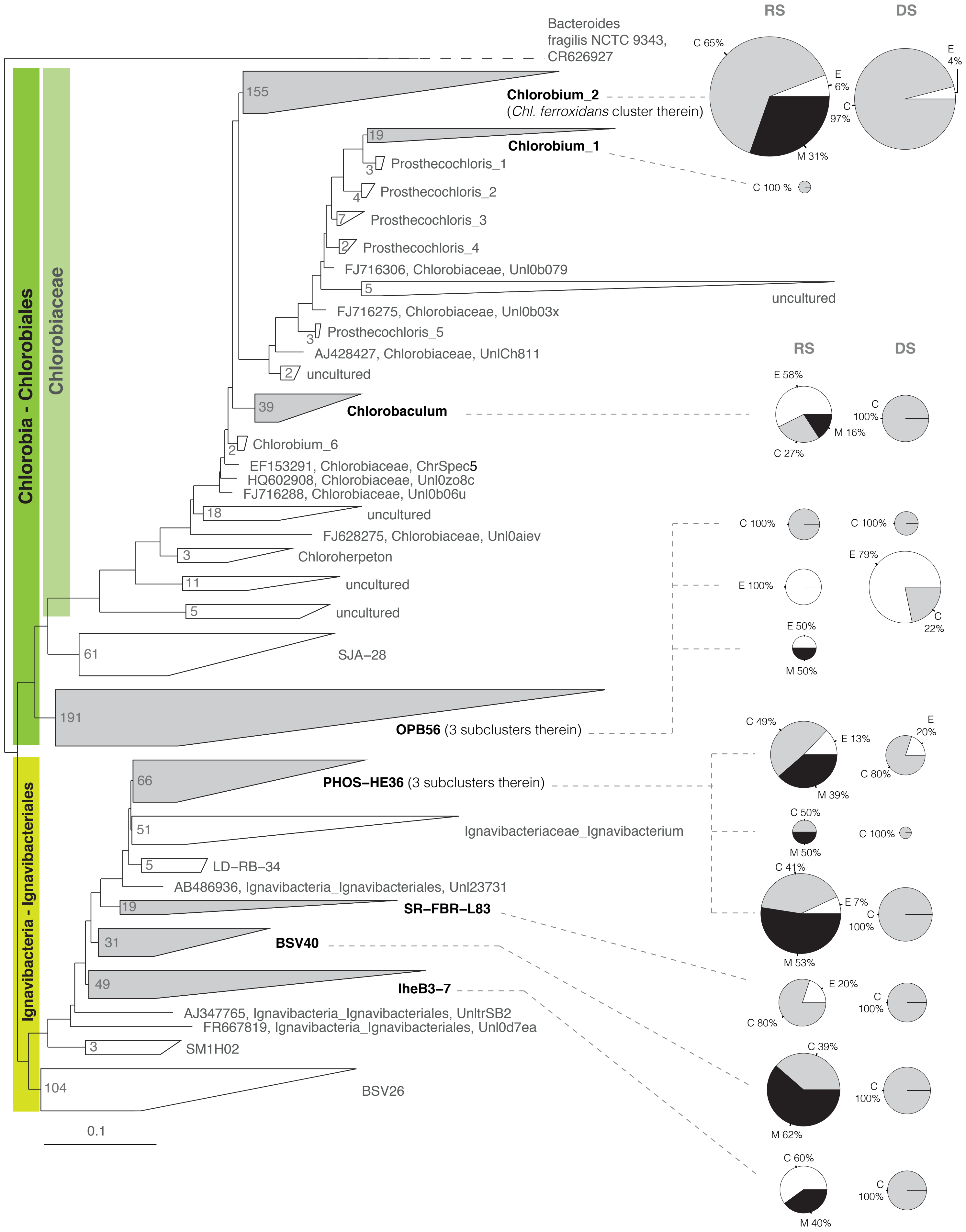

Bacteroides fragilis NCTC 9343, CR626927
RS
DS
Chlorobia - Chlorobiales
Chlorobiaceae
155
Chlorobium_2
(Chl. ferroxidans cluster therein)
C 65%
E 6%
M 31%
E 4%
C 97%
19
Chlorobium_1
C 100 %
3
Prosthecochloris_1
4
Prosthecochloris_2
7
Prosthecochloris_3
2
Prosthecochloris_4
FJ716306, Chlorobiaceae, Unl0b079
5
uncultured
FJ716275, Chlorobiaceae, Unl0b03x
3
Prosthecochloris_5
AJ428427, Chlorobiaceae, UnlCh811
2
uncultured
39
Chlorobaculum
E 58%
M 16%
C 27%
C 100%
2
Chlorobium_6
EF153291, Chlorobiaceae, ChrSpec5
HQ602908, Chlorobiaceae, Unl0zo8c
FJ716288, Chlorobiaceae, Unl0b06u
18
uncultured
FJ628275, Chlorobiaceae, Unl0aiev
3
Chloroherpeton
11
uncultured
5
uncultured
61
SJA–28
191
OPB56 (3 subclusters therein)
C 100%
C 100%
E 100%
E 79%
C 22%
E 50%
M 50%
Ignavibacteria - Ignavibacteriales
66
PHOS–HE36 (3 subclusters therein)
C 49%
E 13%
M 39%
E 20%
C 80%
51
Ignavibacteriaceae_Ignavibacterium
C 50%
M 50%
C 100%
5
LD–RB–34
AB486936, Ignavibacteria_Ignavibacteriales, Unl23731
19
SR–FBR–L83
C 41%
E 7%
M 53%
C 100%
31
BSV40
E 20%
C 80%
C 100%
49
IheB3–7
C 39%
M 62%
C 100%
AJ347765, Ignavibacteria_Ignavibacteriales, UnltrSB2
FR667819, Ignavibacteria_Ignavibacteriales, Unl0d7ea
3
SM1H02
104
BSV26
C 60%
M 40%
C 100%
0.1

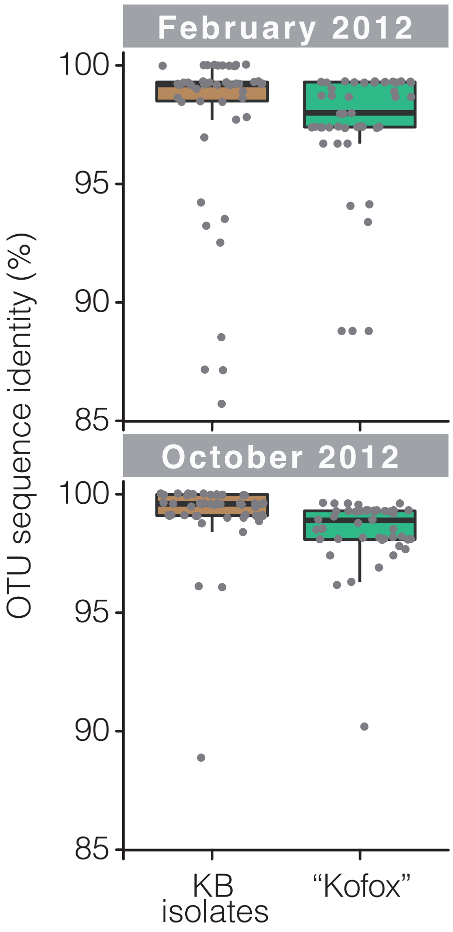
February 2012
October 2012
OTU sequence identity (%)
100
95
90
85
KB isolates
“Kofox”

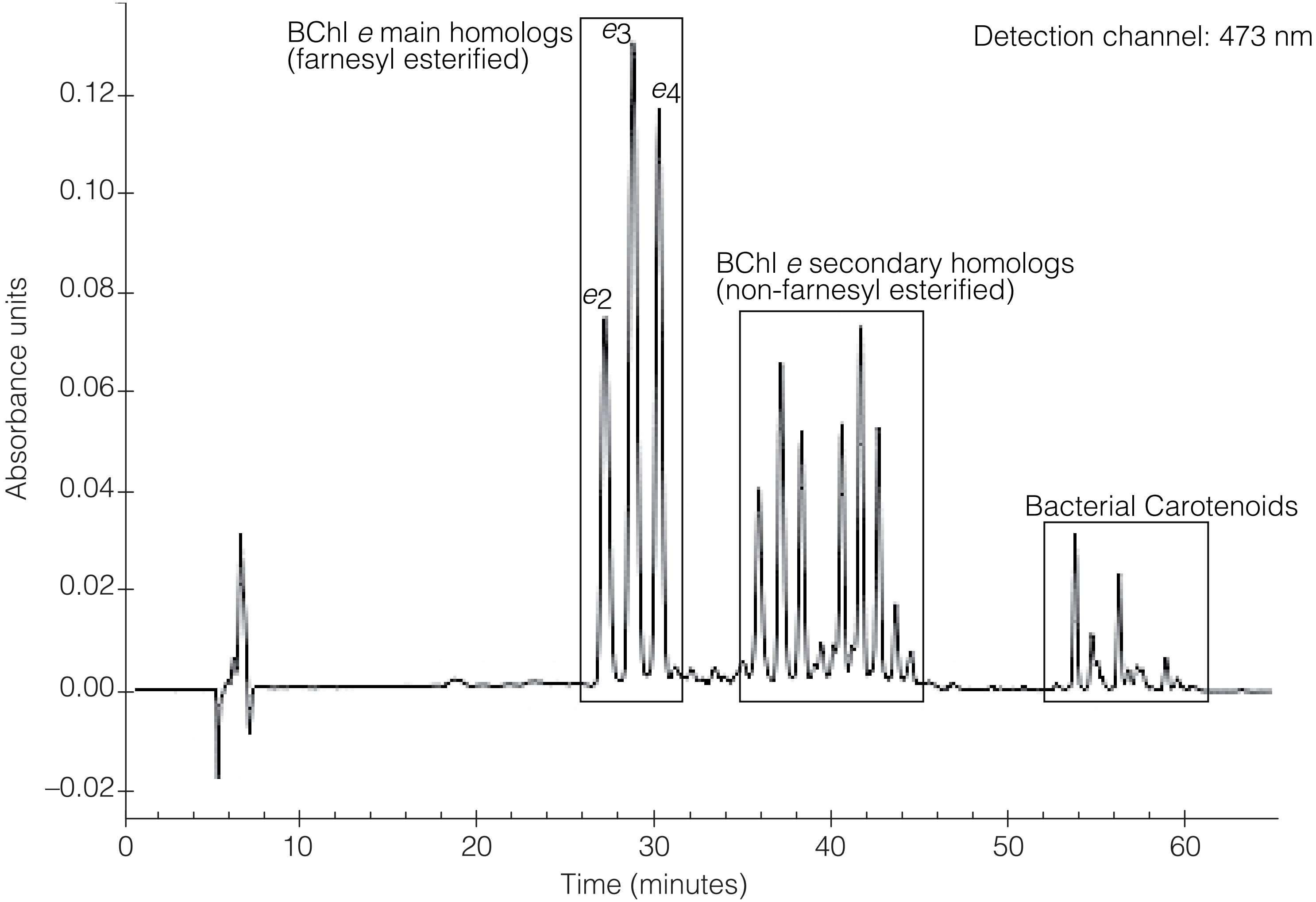

BChl e main homologs
(farnesyl esterified)
e3
e4
e2
Detection channel: 473 nm
BChl e secondary homologs
(non-farnesyl esterified)
Bacterial Carotenoids
Absorbance units
0.12
0.10
0.08
0.06
0.04
0.02
0.00
–0.02
0
10
20
30
40
50
60
Time (minutes)

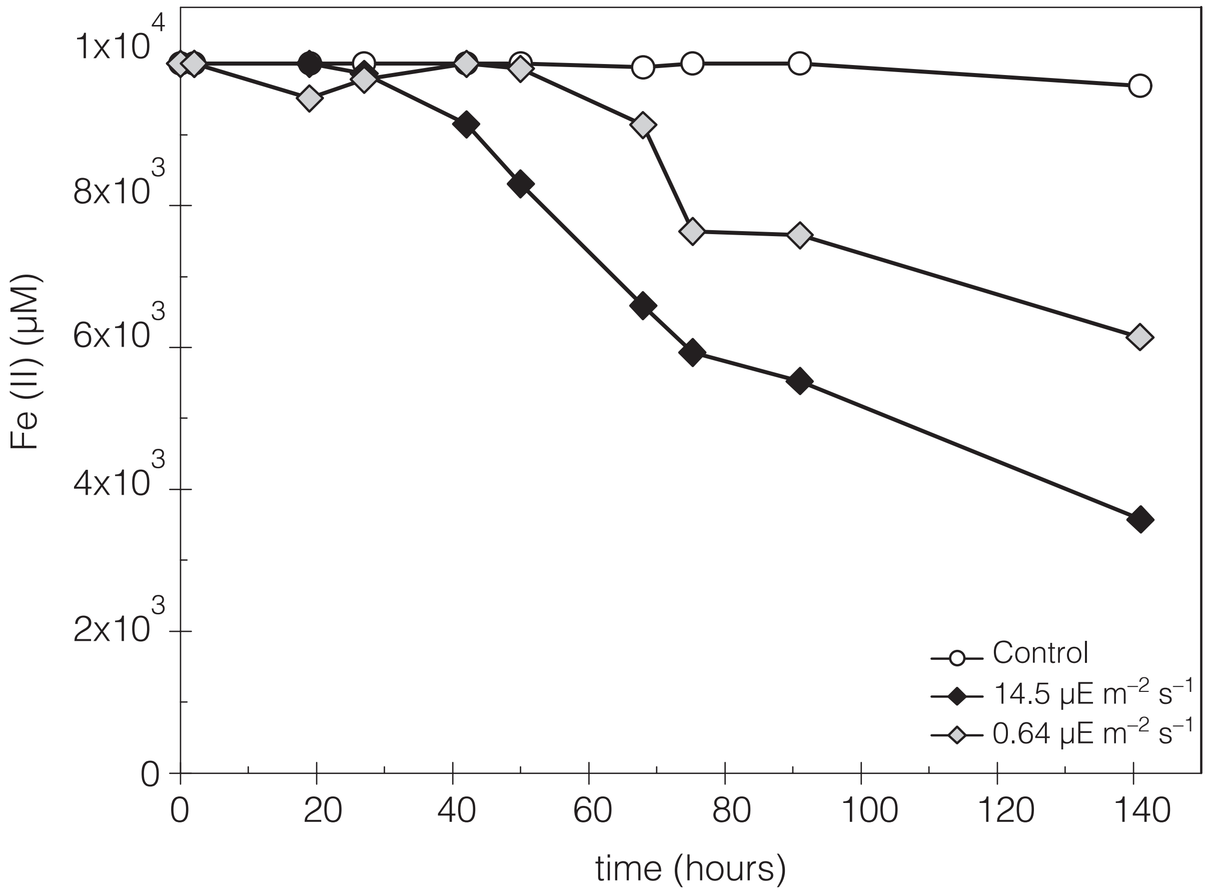

1x10^4
8x10^3
6x10^3
4x10^3
2x10^3
0
Fe (II) (µM)
0
20
40
60
80
100
120
140
time (hours)
Control
14.5 µE m^-2 s^-1
0.64 µE m^-2 s^-1
